# Accelerating Digital Mental Health Research From Early Design and Creation to Successful Implementation and Sustainment

**DOI:** 10.2196/jmir.7725

**Published:** 2017-05-10

**Authors:** David C Mohr, Aaron R Lyon, Emily G Lattie, Madhu Reddy, Stephen M Schueller

**Affiliations:** ^1^ Center for Behavioral Intervention Technologies Department of Preventive Medicine Northwestern University Chicago, IL United States; ^2^ Department of Psychiatry and Behavioral Sciences University of Washington Seattle, WA United States; ^3^ Department of Communication Studies Northwestern University Evanston, IL United States

**Keywords:** eHealth, mHealth, methodology

## Abstract

Mental health problems are common and pose a tremendous societal burden in terms of cost, morbidity, quality of life, and mortality. The great majority of people experience barriers that prevent access to treatment, aggravated by a lack of mental health specialists. Digital mental health is potentially useful in meeting the treatment needs of large numbers of people. A growing number of efficacy trials have shown strong outcomes for digital mental health treatments. Yet despite their positive findings, there are very few examples of successful implementations and many failures. Although the research-to-practice gap is not unique to digital mental health, the inclusion of technology poses unique challenges. We outline some of the reasons for this gap and propose a collection of methods that can result in sustainable digital mental health interventions. These methods draw from human-computer interaction and implementation science and are integrated into an Accelerated Creation-to-Sustainment (ACTS) model. The ACTS model uses an iterative process that includes 2 basic functions (design and evaluate) across 3 general phases (Create, Trial, and Sustain). The ultimate goal in using the ACTS model is to produce a functioning technology-enabled service (TES) that is sustainable in a real-world treatment setting. We emphasize the importance of the service component because evidence from both research and practice has suggested that human touch is a critical ingredient in the most efficacious and used digital mental health treatments. The Create phase results in at least a minimally viable TES and an implementation blueprint. The Trial phase requires evaluation of both effectiveness and implementation while allowing optimization and continuous quality improvement of the TES and implementation plan. Finally, the Sustainment phase involves the withdrawal of research or donor support, while leaving a functioning, continuously improving TES in place. The ACTS model is a step toward bringing implementation and sustainment into the design and evaluation of TESs, public health into clinical research, research into clinics, and treatment into the lives of our patients.

## Background

Mental health problems are common [1] and present a tremendous societal burden in terms of cost, morbidity, quality of life, and mortality [2,3]. About two-thirds of people with mental health problems want some form of psychological treatment [4-8]. However, most people experience barriers that prevent access to such treatments [9,10]. Furthermore, there are not enough mental health professionals to meet the needs of the population [11]. To deliver mental health care to all patients who need and desire it, the care system will require services that can be delivered cost effectively, remotely, and in settings where people most frequently receive care such as primary care or community social services [11].

Digital mental health technologies such as Web-based and mobile apps are frequently cited as potential methods of extending effective care in a cost-effective manner [12]. Randomized controlled efficacy trials have consistently demonstrated that these technology-based tools, when coupled with support from a coach or clinician, can produce benefits similar to those seen for psychological treatments [13-18]. Yet for all the indications that digital mental health interventions can work, evidence is emerging that such interventions have not been effective in routine care settings. The largest implementation trial to date, conducted in England’s National Health Service, compared two of the best-established coach-supported Web-based interventions (Beating the Blues and MoodGym) for depression with treatment as usual in primary care and found no significant benefits [19]. Patients did not engage with the Web-based treatment programs and even stopped answering the coaches’ calls. This mirrors the experience of large health care organizations in the United States such as Kaiser Permanente, which have unsuccessfully tried many times to implement well-known digital mental health interventions such as Beating the Blues [20]. Similar to the experience in England, patients did not use them, coaches and providers were uncertain how to engage patients, and it was unclear how to integrate these treatments into the care system.

The aim of this paper is to describe the challenges that face the field of digital mental and behavioral health research related to this research-to-practice gap. We focus on the challenges of developing and sustainably implementing technology-enabled treatment services within care systems and not on standalone products such as those available through app stores. While digital behavioral health interventions such as those targeting diet and exercise also face substantial implementation challenges [21,22], the technology, commercial, and clinical contexts are quite different relative to mental health. Thus, while this paper may have relevance for the broader field of technology-enabled behavioral interventions, we begin here with a narrower focus on mental health. We propose a clinical research model that integrates several methodologies to rapidly move from initial design through to implementation and sustainment within care systems.

## Challenges

The challenges can be grouped into the three Ds: duration of the research process, design, and denominator of recruitment [20].

### Duration of Research Process

In medicine it can take up to 17 years to move 14% of original research into patient care [23]. Clinical science has developed frameworks for evaluation that aim to protect the interests of stakeholders, including patients, providers, and payers, by verifying the efficacy, effectiveness, and safety of interventions. In psychology, models are based on the US Food and Drug Administration’s prescribed phases for the evaluation of pharmaceuticals. These five phases are (1) intervention generation and refinement, (2) efficacy in research clinics, (3) efficacy in community settings, (4) effectiveness, and (5) implementation [24,25]. Other models exist such as the deployment-focused model [26] that are less linear and more focused on effectiveness but have a similar number of steps.

These methods have made the process of bringing research into practice both inefficient and ineffective [27-30]. These problems are even more pronounced in digital health, where the pace of technological innovation is rapid and consumer expectations about the capabilities of technologies are rapidly evolving [31-33]. A great deal can change with technology in 17 years: iPhones were first launched in 2007 with Android following in 2008, and today smartphones are the dominant method of accessing the Internet for many Americans [34]. Research in digital health intervention must be translated rapidly into practice to avoid validation of interventions that rely on obsolete technologies [35].

### Design

The design of digital interventions has been lacking in a number of ways. First, most digital mental health technologies, which have been evaluated through trials, have largely been designed top-down [36-38], with experts specifying interventions consistent with behavioral strategies derived from evidence-based treatments. The resulting interventions have been primarily Web-based and predominantly psychoeducational via text or video, with some simple interactive tools for common evidence-based practices such as tracking of relevant symptoms and scheduling and monitoring of targeted behaviors. While usability testing has gained currency in recent years [35], the design of these digital interventions has generally not included input from end users. Thus, the field has generally designed interventions to try to get people to do what experts believe is beneficial and has paid far less attention to what users want or how to fit tools into the fabric of users’ lives.

Second, when design occurs, it focuses nearly entirely on the technology components. The evidence, however, indicates that digital interventions require some human support to obtain substantive and reasonably reliable benefits [16], and indeed, optimization of the design of human support services may have a greater impact on clinical outcomes than does the design of the technologies [39]. Thus, digital mental health interventions are essentially sociotechnical systems, which we call technology-enabled services (TESs) to emphasize that this patient-supporter interaction is at least as critical as patient-technology and supporter-to-technology.

Finally, implementation and sustainment are rarely considered during the design of TESs. Most TESs tend to be developed by academic or commercial teams outside of the settings where they would eventually be deployed [40]. Thus, designs do not include requirements, processes, and constraints of routine care settings such as designing and implementing referral processes; managing coaching or support in the context of a clinic, practice, or agency; communication needs among providers; the needs of administrators; or the information technology needs.

### Denominator problem

Most trials of digital interventions have recruited from very large pools of potential participants active on the Internet, raising questions of generalizability [20]. For example, one recent study evaluating a coached Web-based intervention had a relatively clear denominator, recruiting 406 participants from a pool of 8.7 million insurance plan members (0.00047% enrollment) over a year [41]. The ability to fill trials from an extraordinarily large pool of potential participants means that those recruited are likely unique and rare individuals who are motivated and willing to engage with developed TESs [20]. This introduces biases limiting the generalizability of both the research findings and the TESs themselves, thereby reducing the likelihood of successful implementation and sustainability.

## Accelerated Creation-to-Sustainment Model

**Figure 1:**
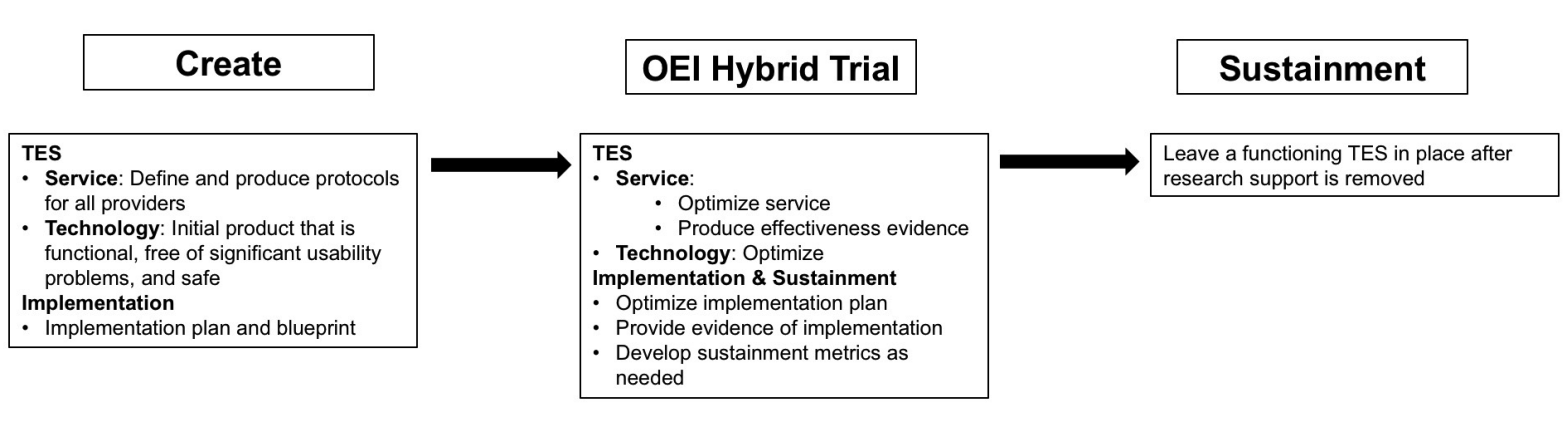
Aims for each development target in each phase.

**Figure 2:**
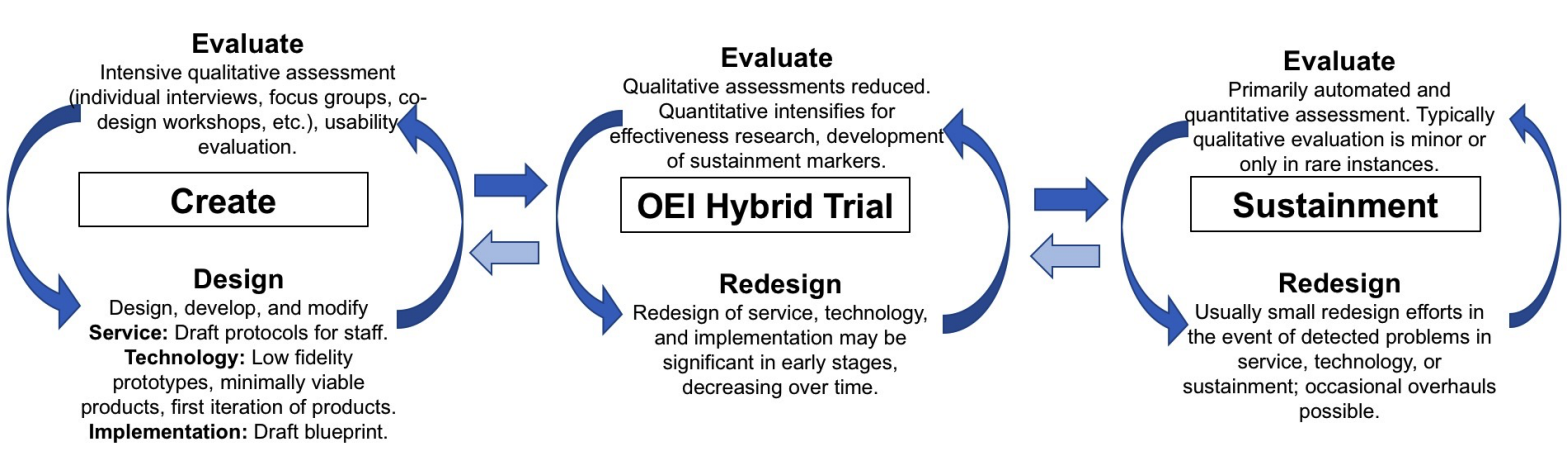
Iterative evaluative and design functions at each phase.

What is needed to overcome these challenges is an end-to-end approach that can move rapidly from the initial design stages of a TES through to implementation and sustainment while ensuring that factors critical for the entire process are evaluated and addressed. We propose the Accelerated Creation-to-Sustainment (ACTS) model that builds on existing methodologies, including human-computer interaction, implementation science, and trial methodology and aims to develop and sustainably implement a TES. As displayed in Figure 1, the ACTS model has three targets across three research phases. First, the Create phase aims to produce protocols for the service, an initial, functional version of the technology that supports that service, and an implementation blueprint. The Trial phase builds on an effectiveness-implementation trial [42] to evaluate the efficacy of the TES and implementation plan, optimize the TES and implementation plan, and development metrics for sustainment. The Sustainment phase withdraws research support gradually, leaving a sustainable TES in place. Figure 2 shows the iterative functions (evaluate and design) that occur for the three development targets (TES service and technology components and implementation and sustainment procedures) across each phase.

## Functions and Targets

The ACTS model is made up of two basic iterative functions, design and evaluate. These functions persist across each phase although the specific methods employed may vary. This stands in contrast to other methodological models for the development and evaluation of digital technologies for behavior change, which argue that design precedes assessment and sharing (eg, the Integrate, Design, Assess, and Share process [43]).

These functions are applied to three development targets relevant to digital mental health, each of which is critical to the ultimate goal of a sustainably implemented TES. The goal of creating a TES requires definition of the service and the technology. The service refers to the clinical goals, behavioral strategies, and expected roles of each of the actors in the intervention (including the patients, care managers, and any clinicians), much as a treatment protocol might for a standard behavioral intervention. The technology refers to the technologies that enable the service. While much of the research has been on the patient-facing side of technologies, as we shift toward viewing the technology as supporting the service, the design and evaluation of technologies will have to be extended to interfaces and systems that support all stakeholders, including patients, care managers, clinicians, and administrators. Implementation and sustainment targets produce processes that facilitate successful delivery of the TES within a treatment setting and its continued use over an extended period, even after any research support is terminated [44,45].

Virtually every mental health treatment technology has been designed in academic labs or by commercial developers, typically outside of clinical settings. As has been seen generally in health research, the chasm between research and implementation is wide and not easily bridged [29]. While it is difficult to overcome the research-to-practice gap for behavioral interventions generally, the technological components add additional cost and complexities to adapting the intervention to fit the context. To succeed in creating an effective, implementable, and sustainable TES, design and research must take place in the settings where they are expected to be deployed. Implementation and sustainment strategies must be designed into the TES from the very beginning [46].

## Phases

The ACTS model has three general phases that rapidly carry the project from initial conceptualization to a sustainable TES. These are the Create, Trial, and Sustainment phases. The aims for each of these phases, displayed in Figure 1, reflect the product or output for that phase.

### Create Phase

The first phase is focused on the development of the TES (both technology and services components) and implementation strategies. Although much has been written about the importance of “designing for dissemination” or “starting early for sustainment” [47], implementation and sustainment strategies are rarely developed alongside the services they are intended to support. A clear definition of the service is critical, because this frequently involves new roles and functions and potentially new professionals who can help support technologies and services [48]. This phase employs user-centered design [49,50] that emphasizes deep engagement with key stakeholders (eg, patients, providers, administrators) and their organizational and social contexts to produce a well-designed TES [51].

The aim of the Create phase is to develop a service protocol, a technology, and an implementation/sustainment plan that is ready for first deployment. Success at the end of the Create phase would include having a (1) clearly specified service delivery model that includes basic protocols for any staff involved in TES service provision, (2) a set of enabling technologies that are safe and have preliminary evidence that they meet criteria for technical and functional reliability (technical reliability means that the software and hardware perform consistently according to specifications; by functional reliability, we mean that users can use the technologies to perform the intended actions), and (3) a set of contextually appropriate implementation/sustainment strategies described in an implementation blueprint that are compelling, easy, and reliable to use. We emphasize that the TES and implementation plan do not need to be final products. Both to accelerate the process and avoid wasteful over-optimization early in the process, the goal of the Create phase is to produce a minimally viable TES and implementation plan that are expected to serviceable, which can then be optimized in the next phase.

To meet these aims, design processes are used that we refer to here as user-centered design but which have also been referred to as cooperative design, participatory design, and contextual design [52-54]. This user-centric approach to development recognizes that the success and adoption of technology depends on people’s experiences and the ecosystem’s support of that technology.

At the core of user-centered design is a focus on collaboration between the designers and stakeholders to provide stakeholders with low-effort ways to inform, contribute to, and interact with technologies during the formative processes. User-centered design’s systematic approach to integrate information and iteratively improve products and services is useful to the scientific process of ensuring replicability and generalizability. User-centered design usually begins with acquiring information from key stakeholders to understand user requirements that can contribute to an initial design document [55]. This design document leads to low-fidelity prototypes, which are designs on paper, videos, or nonfunctional tools that contain key functions of interests. Such prototypes are useful because they can be presented to testers for evaluation and can be redesigned based on that evaluation. This allows assumptions to be tested early and makes use of the stakeholders’ expertise in the domains in which they will use and support technologies in practice. This cycle continues with progressively more fully functioning, higher fidelity versions until the product is functional and free of major design flaws, at which point it is ready for initial implementation.

User-centered design typically begins with acquiring a basic understanding of the goals, challenges, and motivations of each of the stakeholders, particularly in relationship to the management of mental health conditions. In addition, an understanding of the affordances and constraints associated with organizational factors is important for developing the implementation blueprint.

Initial evaluation of user requirements, particularly when little is known, can begin with individual interviews and focus groups to understand the needs, wants, and limitations of key stakeholders [56,57]. However, gathering information about requirements often involves watching in addition to asking. Workflow observations can help illuminate important TES features (both technical and coaching) and how TES might be integrated into the larger care context. These observations can help identify the organizational barriers that users would face in using the TES that may not emerge from interviews or focus groups [58,59].

After a basic understanding is acquired, more interactive methods can deepen an understanding of the kinds of design elements that may prove useful. For example, codesign workshops bring together the researchers and stakeholders to help representative end-users begin designing their own solutions that address their needs [50,60,61]. Stakeholder participants can draw sketches of features or tools that may be of interest to them [62], describe potential services or interactions, and even interact with paper prototypes [63]. The role of the researcher is to help participants translate these solutions into effective design constructs [64]. At the core of the codesign concept is the idea that stakeholders themselves are best positioned to articulate these solutions.

In user-centered design, as user requirements become clear, the iterative process of design, evaluate, redesign begins. This iterative process refines the design ideas with collaboration between the designers and stakeholders that eventually will be developed into the technologies, services, and implementation strategies [50,64]. Task analysis is one method, in which stakeholders are presented low-fidelity prototypes, which are simple versions of a tool with no real functionality, and asked to complete tasks that are part of the service design. These actions are observed, recorded, and analyzed. Based on errors or problems, corrections can be made to the design of the prototype or to the service protocol, and testing begins again [65]. Heuristic evaluations require experts who judge the compliance of prototypes with recognized usability principles [66]. Cognitive walkthroughs ask stakeholders to work through a series of tasks, which can be useful for evaluating implementation protocols [67]. Once the major flaws in the service protocol, technology, and implementation plan have been eliminated, the TES can progress to the trial stage. We note that because further refinements can be made during the trial, it is not necessary to test to perfection. It is preferable to get into the field as quickly as possible.

### Optimization, Effectiveness, and Implementation Hybrid Trial Phase

The second phase is focused on the evaluation of the TES and implementation strategies with regard to clinical goals. The objectives in this phase are to optimize the TES so that it meets its clinical objectives and is usable by all stakeholders, evaluate its effectiveness, and successfully implement the TES in the setting. These objectives are achieved by testing in real-world clinical contexts rather than research settings. Traditional methods of evaluation would require a sequential, phased series of trials to separately achieve each goal [24]. The ACTS model argues that Optimization, Effectiveness, and Implementation (OEI) studies can occur simultaneously in an OEI Hybrid trial. Optimization extends the Create phase from early testing to longitudinal, iterative evaluation and redesign in the context of deployment, continuing until all major problems have been identified and resolved. Effectiveness is evaluated to provide evidence that the intended outcomes are achieved, protecting stakeholders’ interests. Implementation is evaluated to ensure that the TES can be seamlessly deployed in the intended setting; any implementation problems result in redesign of the implementation plan. Combining these goals in a single trial structure should dramatically accelerate the rate of translational gains of effective TESs, providing useful information to key decision makers in a far timelier manner.

The OEI Hybrid trial builds on Curran’s work and is similar to the Type 2 trial in which effectiveness and implementation outcomes are tested simultaneously [42], with effectiveness components focusing on patient-level clinical outcomes and implementation focusing on contextual and organizational factors such as adoption, uptake, treatment fidelity, costs and cost effectiveness, and efficiency [45,68,69]. While this condensation of effectiveness and implementation trials can reduce the time from initial concept to delivery, the phased research steps before this (pilots and efficacy) will still likely result in obsolescence in the technological elements of the TES. For this reason, as many have argued, the phase model of treatment development and evaluation, which typically lasts many years, adds insufficient value above that provided by effectiveness trials [28]. The value provided by phase models is not worth the threat posed by technological obsolescence [31].

To accelerate the design-implementation pipeline for TESs we argue that optimization of the TES and the implementation plan using the iterative functions of evaluation and redesign must be integrated into the hybrid trial methodology. A traditional, linear phase model would require that a pilot or field trial be conducted prior to launching a TES in a trial to ensure the intervention and technology is working as intended [24,25]. However, optimization outside the clinical context is of limited benefit because many of the challenges of optimization are related to the deployment context. Thus, we argue that the additional 6 to 12 months spent piloting outside the clinical context is overoptimization that fails to collect necessary contextual information and is therefore superfluous. Optimization of the TES and implementation plan together is required because the TES must adjust to issues that arise from unanticipated contextual and organizational issues. Indeed, such optimization during a trial is so common that it is recognized in the Consolidated Standards of Reporting Trials (CONSORT) eHealth guideline, which requires reporting of changes made to the intervention during the trial [70]. Accordingly, we argue that once the TES and implementation plan meet basic usability requirements and there is reason to be believe the TES is safe and may be useful to patients, the OEI Hybrid Trial can begin.

Optimization can continue during the Hybrid Trial, relying on the dynamic, iterative processes of user-centered design. Optimization is facilitated by the articulation of clear specification for objectives, constraints, and design variables [71].

Objectives of optimization are the outcomes that are to be maximized by the TES and the implementation plan. Typically, the primary objective of a TES is a reduction in clinical markers (eg, symptoms of depression). Secondary objectives might include use variables such as communication patterns between the patient and provider. Technology objectives typically include markers of usability including satisfaction, usefulness, ease of use, and absence of errors [72]. Implementation objectives may include measures of penetration, adoption, and cost [45,73].

Constraints refer to conditions that must be met for the viability of the service, technology, and implementation. These constraints can be identified and defined during the Create phase as information regarding the needs and limitations of the stakeholders and organization is accumulated.

One class of constraints relates to methods required to ensure that optimization does not threaten the internal validity of the Hybrid Trial. Making changes to the TES or implementation strategy flies in the face of traditional methods drawn from trial methodology based on pharmaceutical trials in which the active agent is fixed. While it is questionable how locked down behavioral interventions have ever been in randomized controlled trials, technology-based interventions demand changes to fix bugs and prevent obsolescence. The Trials of Intervention Principles method offers ways of integrating continuous quality improvement into the trial while imposing constraints on the scope of design changes to ensure that the TES and implementation plan remain conceptually consistent over the course of the study [32]. A clear framing and operationalization of the principles being evaluated serve as constraints that limit the types of changes that can be made through optimization. Documentation and reporting of changes creates transparency. For example, an intervention focused on treating depression in primary care using care coordinator–supported mobile tools could not suddenly change the outcome or shift to a fundamentally different intervention model (eg, psychotherapy or pharmacotherapy). If an aim is to examine a TES administered by care managers, shifting to mental health providers would constitute a new treatment and trial. On the other hand, some alterations to the patient-facing or care manager–facing tools or the frequency of contacts may be within scope.

Constraints may also be imposed by the organization. For example, the amount of time available from staff, allowable operations in a clinic, or technological requirements may all constitute constraints. New constraints may also be identified during the OEI Hybrid Trial as unintended consequences are unearthed. For example, a TES may end up serving as a conduit into traditional services or help retain patients in treatment, thereby aggravating care capacity problems [74], resulting in constraints to manage the emergent problem.

Design variables refer to those specifications of the service, technology, and implementation that are modifiable and that may affect the objectives (eg, definition of the service, design of technologies, or implementation blueprint). Optimization seeks to refine the design variables to maximize the objectives without exceeding the constraints.

Relative to the Create phase, where information gathering is intended to result in initial definition and development of the TES and implementation approaches, information gathering in the optimization part of the Hybrid Trial phase addresses adjusting the fit for the organizational contexts. This includes responding to unanticipated problems and opportunities encountered in implementation, removing unnecessary components, and improving processes and functionality. Information for optimization, particularly for objectives and constraints, may be collected through defined methods such as the acquisition of data on clinical outcomes, system use, and stakeholder satisfaction.

Allowing iterative processes of evaluation and design to occur during the trial phase supports ongoing learning to continuously adapt and improve the TES and its implementation. The TES optimization evaluation can employ use data and outcome data collected for the effectiveness and implementation trial as well as semistructured user feedback interviews. Numerous methodologies are available to support this learning and optimization including A/B testing [75], continuous evaluation of evolving behavioral intervention technologies [76], multiphase optimization strategy [77], and trials of intervention principles [32]. The idea of changing an intervention during the trial is generally accepted in digital mental health research, and indeed, the CONSORT eHealth guideline includes the requirement of reporting these changes in the publication of trials [70]. Methods of managing, constraining, and documenting the changes have been articulated that can preserve the validity of the trials necessary to generalize to new settings [32,78]. One would expect that optimization efforts would be more intensive at the beginning of the OEI Hybrid Trial and would diminish as problems are identified and corrected.

### Sustainment Phase

The final phase tapers and removes research infrastructure and support for a TES that has met the aims of the Hybrid Trial phase [44]. Sustainment refers to the continued use of an intervention in a manner that brings benefits after this support is removed [45]. Research teams can play a role in this tapering to help facilitate sustainment processes (eg, ensuring the collection and accuracy of data and supporting analysis). However, eventually these roles should be assumed by clinical staff, allowing researchers to recede into the background and leaving the organization to drive a sustainable TES without assistance. The aim of this phase is not just to study sustainment but to leave the health care site with a TES that can function without external research support and continue to adapt to problems that might arise.

Even when initial implementation is successful, sustainment has rarely been examined and, when it has, a lack of sustainment is evident [79]. There are numerous potential threats to sustainment, particularly for interventions that rely on human services in the context of complex, multilayered systems including changes within the organization (eg, staff turnover, change in service organization, leadership, information technology infrastructure) or the larger context (eg, consumer technologies, patient attitudes and preferences, funding models) [80,81].

Given the challenges of sustainment, it is unlikely that a “set it and forget it” model of sustainment will be successful. Thus, the iterative cycle of evaluate and redesign, both to address problems that arise and adjust to changing needs, demands, and contexts, is likely required into the Sustainment phase. Emerging research suggests that sustainment requires active components to identify and correct potential problems and continue to improve and adapt the TES to meet changes in the organization, patient population, and larger context [80,81]. More recent conceptualizations of sustainment have incorporated features of learning health care systems that mirror our evaluation and redesign functions, using data inputs to continuously monitor and dynamically correct and adapt the intervention [35,46,82].

Data collection for dynamic sustainment models must be as effortless as possible so as not to add burden to care systems that are already functioning at or beyond capacity. Fortunately, TESs provide an increasingly large amount of unobtrusively collected data that can be used to monitor the health of the intervention along the entire pipeline. For example, penetration can be monitored by the flow of referrals through various stages of the TES. In a primary care setting, such metrics might include the proportion of appropriate patients referred to TES, proportion of those referred contacted by TES staff, proportion of those contacted who initiated TES treatment, and proportion of those who initiated TES treatment who complete it. Effectiveness can be monitored through symptom measures that are commonly used in TESs for self-monitoring as well as any other electronic records that may be available. Fidelity of the provider to the service protocol is an important component of sustainment. Fidelity can be monitored by having structured protocols represented within provider-facing technology components such as a digital checklist for specific provider actions during patient interactions. Patient satisfaction and quality of use can be monitored with qualitative and quantative feedback from patients and technology use data.

These largely unobtrusive sustainment metrics could be developed for the specific TES during the OEI Hybrid Trial. Fidelity to service models (eg, frequency and timing of contacts) can be assessed in conjunction with the supervisory functions that are important to the success of sustainment, providing benchmarks against which passively collected data from TES use can be used to develop empirically derived fidelity markers [83]. Methods for assessing fidelity will likely become increasingly unobtrusive as natural language processing capabilities improve, enabling automated extraction of fidelity markers from phone calls and messaging [84].

Redesign efforts at the Sustainment phase should be minor. Once a TES is fully implemented and integrated, substantial redesign and redevelopment of technical components can be many times more complex and costly compared to identifying and optimizing during earlier phases [85]. Similarly, major changes to services and workflows are disruptive and challenging to put into practice once integration of TESs is complete.

While the cost and complexity of major redesign efforts during Sustainment is high, all systems eventually require substantial redesign to meet changing needs, preferences, and contexts. In such instances, reintroduction of optimization methods from the OEI Hybrid Trial may help to understand context-specific objectives and constraints to alter the design. This is represented by the recursive arrows in Figure 2.

## Hypothetical Example

It is often helpful to have a concrete example of a model to help convey concepts that can be difficult to grasp through abstract descriptions and discussions. Unfortunately, given our presentation of the ACTS model is an initial proposal, no example currently exists. However, in Table 1 we present a brief hypothetical example of the development and implementation of a TES for a primary care setting. We provide examples of methods and evaluation at each stage, along with the resulting products and possible outcomes.

**Table 1:** Hypothetical example of the Accelerated Creation-to-Sustainment model for a technology-enabled service to treat depression in primary care.

Phase and stage		Methods and evaluation	Design products and outcomes
**Create**			
	User-centered design: TES^a^	Individual interviews with primary care physicians and staff Representative patients, administration in psychiatry, primary care, and medical director's office Codesign workshops with patients, care managers, primary care physicians, mental health specialists, and researchers and developers Quality assurance conducted with staff examining technical and functional reliability Usability testing with patients and care managers	Sketches and paper prototypes Design document and specifications A functioning TES that can be delivered by care managers Patient-facing tools are mobile app-based; care manager tools are computer-based TES has received only limited longitudinal testing in target population but is minimally viable
	User centered design: Implementation plan	Contextual evaluation Individual interviews with primary care physicians and staff, representative patients, and administrators Workflow observations of physicians and care managers Cognitive walkthroughs of typical clinical tasks	Implementation hassle map that outlines where hassles and breakdowns in care occurs Implementation plan defined Plan leverages existing processes in which patients assessed in primary care are referred to TES Processes are defined for TES support, supervision, quality, and safety monitoring Patient population and TES use cases defined
**OEI^b^ Hybrid Trial**			
	Trial design	Blocked randomized controlled trial	Clinical and services outcomes Improvements in symptoms of depression; use of TES by patients and care managers Patients referred to, initiating, and completing TES Efficiency of care managers at providing services
	Optimization	Evaluation of outcomes, use data, and periodic user feedback interviews As problems in TES are identified, changes made and logged	Change log with changes described TES and implementation plan updated iteratively until they function well
	Evaluation	Symptom evaluation by self-report from all patients at all sites meeting criteria for depression TES use data monitored Care management fidelity monitored using random ratings of recorded calls and communication logs Implementation monitored Medical and mental health costs monitored and compared across sites All metrics evaluated over time to explore changes in effectiveness	Changes in symptoms of depression; use of TES by patients and care managers
	Implementation	Qualitative and quantitative evaluation of implementation TES use data monitored Care management fidelity monitored using random ratings of recorded calls and communication logs Implementation monitored Medical and mental health costs monitored and compared across sites All metrics evaluated over time to explore changes in implementation Interviews with system stakeholders (eg, care managers, primary care physicians and staff, administrators) to assess perception of benefit or barriers	Fidelity scores of services provided by case managers Service utilization and costs of services by patients Suggested enhancements for implementation (eg, training, deimplementation of ineffective elements)
**Sustainment**			
		Unobtrusive markers Ongoing monitoring of symptoms through within treatment evaluation, system usage by patients and care providers, markers of fidelity (eg, pattern of care manager outreach, outcomes across care managers), referral patterns	Ongoing benefits of TES system A functioning TES that is supported by clinical staff and feeds appropriate and actionable information back to staff, providers, and administrators

^a^TES: technology-enabled services.

^b^OEI: optimization, effectiveness, and implementation.

## Managing Potential Failure Points and Problems

There are potential risks and failure points within each phase. The user-centered design process in the Create phase should result in a design and initial product. The primary risk during the design phase is that design challenges will take longer than expected to address. For example, aligning the objectives and preferences of patients, providers, and administrators may prove more challenging than initially expected. There are several potential failure points during the OEI Hybrid Trial. First, it is possible that design and implementation cannot be optimized sufficiently to create a workable solution. For example, it is possible that not enough people are recruited into the trial, which would reduce the amount of information gained and provide insufficient resources or rationale to continue optimization procedures. As we note above, this would be an indicator of a problem in design of the TES or implementation plan and could require returning to the Create phase (represented by the recursive arrows in Figure 2). It is also possible that even with good optimization and implementation, the intervention is simply not effective, which could result in a return to user-centered design in the Create phase or abandonment of the effort. Assuming successful implementation and evidence of effectiveness, the program can move forward to the Sustainment phase. There are numerous potential contributors to a failure of sustainment of a TES that has made it through the OEI Hybrid Trial, including lack of support and guidance of staff and care managers, lack of resources, lack of buy-in from senior management, or failure to detect and address problems that arise over time [83,86,87].

We recognize that the accelerated process evaluation, in particular moving a TES into a trial phase earlier in the optimization process, opens the possibility of increased risk to participants. Such risk is low; negative outcomes among patients receiving digital interventions is rare, is lower than in control conditions, and does not appear to occur at rates higher than standard treatments [88]. However, while such risk is low, it is important to evaluate and guard against. Monitoring for patient risk should occur in all phases, with particular attention paid to the possibility of iatrogenic effects during the Create and early OEI Hybrid Trial phases. Such monitoring should use both quantitative and qualitative methods to detect potential iatrogenic effects (eg, “Did you experience any problems or negative consequences in your treatment?”) and to obtain more detailed information when such effects are detected [89]. Introducing any new service into a care setting has the potential to introduce other negative effects at the provider and system perspective as well. For example, TESs could produce such a wealth of information that providers and systems are not able to process it into actionable guidance when appropriate (such as safety alerts in the case of suicidal patients).

## Conclusion

There is an enormous research-to-practice gap in digital mental health, with strong and growing evidence from efficacy trials over more than 15 years yet virtually no successful and sustainable implementation. This failure is due to many factors. Our research models are exceedingly reductive, compartmentalizing aims into individual research programs (such as phase models) to answer isolated questions of design, intervention refinement, efficacy, effectiveness, implementation, and sustainment. This is not only inefficient but also ignores the intricacy of delivering TESs involving rapidly changing technological environments into the varied and complex circumstances of individual patients’ lives.

The ACTS model provides a framework for accelerating research and integrating design, evaluation, and sustainable implementation into a unified effort. Evaluation in the Create phase is intensive and qualitative, becoming more quantitative in the Trial phase, and finally leaning heavily on pragmatic methods such as unobtrusive, largely automated measurement in Sustainment. Design flexibility is maximal in the initial design phases and becomes increasingly hard to change and adjust as the TES becomes developed, deployed, and integrated into care settings. It is imperative that we build implementation and sustainment into the design process from the very inception, when there is maximum flexibility. This is especially true when technology is involved; it is far more cost effective to adjust and fix design problems early as opposed to once the technologies and services are in place [90].

While many of the components of this framework, including user-centered design, hybrid trials, integration of optimization and evaluation, and sustainment, have previously been articulated and applied in many contexts, they have not been put together in a single organized model. This paper is intended as a draft of a general blueprint for a new, expedited approach to research in TESs. We would expect and welcome disagreement and refinement. However, we can no longer afford to consider clinical research as divorced from public health. In a world of rapidly evolving technologies, we can no longer wait more than a decade to move research into practice. The ACTS model is a step toward bringing implementation and sustainment into design and evaluation, research into clinics, public health into clinical research, and treatment into the lives of our patients.

## Abbreviations

ACTS model: Accelerated Creation-to-Sustainment model
CONSORT: Consolidated Standards of Reporting Trials
OEI Hybrid Trial: Optimization, Effectiveness, Implementation Hybrid Trial
TES: technology-enabled service
UCD: user-centered design
